# Waterborne Antifouling Paints Containing Nanometric Copper and Silver against Marine *Bacillus* Species

**DOI:** 10.1155/2022/2435756

**Published:** 2022-02-15

**Authors:** G. M. Loredo-Becerra, A. Durán-Almendárez, A. K. Calvillo-Anguiano, I. DeAlba-Montero, L. O. Hernández-Arteaga, F. Ruiz

**Affiliations:** ^1^Institutional Doctorate in Engineering and Materials Science, Autonomous University of San Luis Potosí, San Luis Potosí, S.L.P., Mexico; ^2^Faculty of Sciences, Autonomous University of San Luis Potosí, San Luis Potosí, S.L.P., Mexico; ^3^Intercultural University of San Luis Potosí, San Luis Potosi, Mexico

## Abstract

Due to the concern to find an alternative to reduce the colonization (microfouling and macrofouling) or the biocorrosion of surfaces submerged for long periods in water, we evaluated the antifouling activity of a commercial paint added with silver nanoparticles (AgNP's) and copper nanoparticles (CuNP's), beside copper-soybean chelate, by electrolytic synthesis, using them in low concentrations (6.94*E* − 04 mg Ag g^−1^ paint, 9.07*E* − 03 mg Cu g^−1^ paint, and 1.14*E* − 02 mg Cu g^−1^ paint, respectively). The test for paint samples was carried out by JIS Z2801-ISO 22196 for periods of initial time, 6 months, and 12 months, against three bacterial strains of marine origin, *Bacillus subtilis, Bacillus pumilus,* and *Bacillus altitudinis*. It was possible to demonstrate, according to the standard, that the sample with the greatest antimicrobial activity was the copper-soybean chelate against two of the three strains studied (*B. pumilus* with *R* = 2.11 and *B. subtilis* with *R* = 2.41), which represents more than 99% of bacterial inhibition. Therefore, we considered a novel option for inhibiting bacterial growth with nanoparticles as antifouling additives.

## 1. Introduction

Nanotechnology, being an emerging field where other sciences contribute, generates a large number of new applications based on the novel physicochemical properties acquired by resizing a material to the order of nanometers. Working at the nanoscale, seek to solve current problems in various areas, including materials science, where for example, in the area of paints. Some authors suggest the incorporation of different types of additives, including nanomaterials, to give them new characteristics, as self-cleaning, antibacterial, scratch-resistant, fire-retardant, UV-protected, wood preservation, anticorrosive [1], and antifouling paints. Using antifouling paints prevent damage to boat hulls caused by marine microorganisms [2]. Existing products whose purpose is to prevent the growth and settlement of bacteria, fungi, and algae on ships, the presence of those implies adverse effects for maritime navigation, such as a reduction in speed caused by greater resistance to friction, that leads to an increase in fuel consumption up to 40%. It can also provoke high corrosion rates in the ship's hull, and consequently, the generation of toxic waste and the introduction of exotic species in its wake [3]. In the middle of the 19th century began to formulate antifouling paints. These had copper oxide, arsenic, and mercury oxide in resin binders and showed good effectiveness [4]. In the 1950s, tributyltin (TBT), an organometallic compound, began to be used and for approximately two decades was found in a large percentage of ships around the world [5], but which, despite representing economic benefits, demonstrated at low concentrations, a negative impact on marine organisms [6]. Thus began the search for alternatives to the use of TBT. Some biocides that have replaced it are copper-based compounds such as cuprous thiocyanate and cuprous oxide [7], the latter being the most used in formulations for antifouling paints. In recent research, it has been proposed to use nanomaterials because it would not only help to reduce the concentration of the biocide used but also prevent harmful effects to other marine species and the resistance of some microorganisms to antifoulants that are already on the market. Owing to the antibacterial properties of silver and copper nanoparticles, they are incorporated into some materials to solve current problems [8] such as bacterial contamination [9].

In general, nanoparticles as antifouling agents have been obtained by biosynthesis or chemical reduction methods. According to the study carried out by Inbakandan et al., AgNP's biosynthesis using *Acanthella elongata* produced particles less than 40 nm in diameter, which were evaluated against 16 strains that form marine biofilms. Their results show that the inhibition capacity depends both on the species to be evaluated and on the concentration of nanoparticles used, and they concluded that they obtained a nanomaterial with great bactericidal capacity and antifouling potential [10]. An important finding was presented by Ramasubburayan et al. who found minimum values of minimum inhibitory and bactericidal concentration for their silver nanoparticles, obtained from a biosynthesis mediated by *Bacillus vallismortis*, against encrusting strains, showing promising antibiofilm activities [11].

Copper, due to its properties against microorganisms, including marine organisms, has been used for many years in antifouling paints; therefore, all its structures and compounds have been investigated. Abiraman and Balasubramanian reported a green synthesis of copper nanoparticles decorated with chitosan, with an average size less than 2 nm and whose antifouling activity against marine and green algae showed an antifouling efficacy of between 80 and 95% [12]. Nowadays, there are materials that have attracted interest to investigate them due to their composition and their possible uses in different fields, such is the case of a study conducted by DeAlba-Montero et al. where they demonstrated that there are more options of nanometric materials that have antimicrobial properties, such as copper-soybean chelate. They report growth inhibition against *E. coli*, *S. aureus*, and *E. faecalis* strains at low chelate concentrations [13]. These results generate the concern to use and investigate copper-soybean chelate as a new antifouling additive option.

In addition to green synthesis or biosynthesis for obtaining nanomaterials as proposed by several authors, another suggested method that also presents advantages such as almost zero contamination is the electrochemical method. In this type of synthesis, the nanomaterial can be easily isolated from the precipitate, and it is an easy to replicate method in which the particle size is usually very well defined. Reetz et al. pioneered the electrochemical synthesis of metallic nanocrystals [14]. His method consists of six elementary steps: the oxidative dissolution of the anode, the migration of metal ions to the cathode, the reduction of the ions to a state of zero-valent, the formation of particles by nucleation and growth, the arrest of growth by stabilizing agents, and particle precipitation [15]. One of the main advantages of this method is its great reproducibility and the possibility of modifying different variants in the process. The electrochemical synthesis method for obtaining copper and silver nanomaterials, among others, has been published in the literature; however, they use very long reaction times, high temperatures, more than two or three reagents to activate the solution that acts as electrolytic solution and in some cases, a high voltage [16–21]. In this research, we were able to optimize the synthesis method in such a way that nanoparticles were obtained in a reduced time, at room temperature, with a certain voltage and without the inclusion of a large number of reactives, thus proposing a highly reproducible method for both silver and copper, with a calculated reaction efficiency higher than 90%.

The Organisation for Economic Co-operation and Development (OECD) has emphasized that there are different protocols and relevant standards at a global level that allow the evaluation of articles and products that contain antibacterial additives [22], among which can be listed as follows:

- ASTM D5590-94: Standard Test Method for Determining the Resistance of Paint Films and Related Coatings to Fungal Defacement by Accelerated Four-Week Agar Plate Assay
- Singapore standard SS 345:2015: Specification for algae resistant emulsion paint for decorative purposes
- TESHSA NSI method: A nonsuspended inoculum method for determining the antibacterial activity of coated surfaces

These standards are just some of those that can be used to evaluate antimicrobial products. The vast majority of evaluations are based on qualitative tests, and we can note that these methods determine the activity of fungi and algae, with the exception of the TESHSA NSI method that allows the evaluation with bacteria and being a method that does not belong to any international organization for the standardization of evaluation methods and perhaps is the reason why the Paint Research Association (PRA) in the United Kingdom recommends using the Japanese standard JIS Z2801. Every day more companies use it to validate their products since it is a method supported at an industrial level by paint manufacturing companies [23–25].

We propose the use of silver and copper nanoparticles and copper-soybean chelate as additives to provide antifouling properties to a commercial paint. It is relevant to the fact that these nanomaterials are easily synthesized due to our optimized and simplified synthesis method as well as the preparation of copper-soybean chelate, being a complex containing an organic part, is of great interest for its use in antifouling applications. We suggest that the possible antifouling activity will be due to the activity that have the nanostructured compounds added to the paint, and this will be determined by performing an evaluation according to the Japanese Industrial Standard JIS Z2801-ISO22196 [26], as this is a quantitative and standardized method, which provides reliable and accurate results that improve surface evaluations; however, due to its complexity, it is rarely used. The analyses carried out will allow us to first determine the characteristics of the materials that we are using as antifouling additives, to later observe their behavior as additives in paints and to establish whether the paint prepared with these solutions can be considered as antifouling and/or antimicrobial.

## 2. Materials and Methods

### 2.1. Electrolytic Synthesis of Nanoparticles

#### 2.1.1. Silver Nanoparticles

The reactive materials employed for this synthesis were gallic acid (Sigma Aldrich Co.), nitric acid (HNO_3_ 64–66%), and ammonium hydroxide (NH_4_OH) from Fermont Co. and used without further purification. The electrodes used (silver sheet as anode and graphite as cathode) were placed vertically face-to-face inside the electrolysis beaker. 0.01 g of gallic acid was diluted and added to 150 ml of deionized water and started stirring; later, 0.5 ml of nitric acid and 0.5 ml of ammonium hydroxide were added to the previous solution. A potential was applied by 30 V for 3 minutes, and the resulting solution corresponds to silver nanoparticles (AgNP's).

#### 2.1.2. Copper Nanoparticles

**Figure 1:**
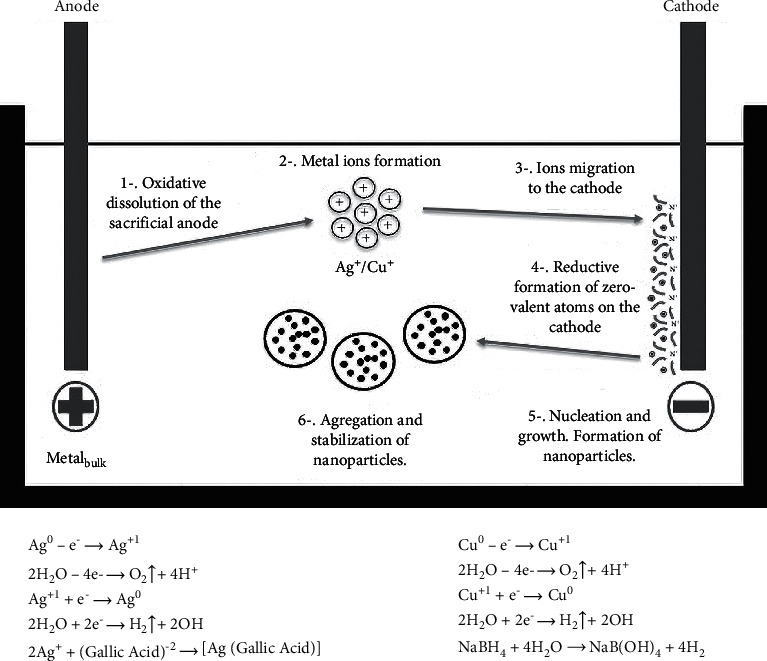
Electrochemical synthesis for the formation of nanoparticles [27].

All the chemicals used in this project were of reactive grade: sodium borohydride (NaBH_4_ ≥98%) and sulphuric acid (H_2_SO_4_ 95–98%) were purchased from Sigma Aldrich Co. and Fermont Co., respectively, and used without further purification. The experiments were carried out in an electrolysis beaker containing a sacrificial copper sheet as anode and graphite as cathode. These electrodes were placed vertically face-to-face inside the cell, 150 ml of deionized water was added, and kept under stirring. Then, 0.5 ml of H_2_SO_4_ was added. Before each experiment, the electrodes were polished and washed with deionized water. The electrodes were activated by a potential of 30 V for 3 minutes. After this time, 0.1 g NaBH_4_ was diluted in 5 ml of deionized water and added to the solution. The precipitate obtained was filtered and washed with a small amount of acetone to prevent the oxidation of the copper nanoparticles (CuNP's). In Figure 1, we can observe a schematic representation of the suggested process for the electrochemical synthesis of nanoparticles [27]. Additionally, we propose the possible chemical reactions involved in the synthesis, which are presented in Figure 1 for copper and silver nanoparticles.

#### 2.1.3. Copper-Soybean Extract Chelate

The solution was prepared according to the method previously reported by Guajardo-Pacheco et al. [28], where 1 g of CuSO_4_·5H_2_O was added to a soybean extract solution; it was kept under magnetic stirring for 15 minutes, and the pH was adjusted to 7 using a 1 M sodium hydroxide solution.

### 2.2. Characterization

Copper and silver nanoparticles formation were confirmed by different structural characterization techniques. Optical absorption spectra were obtained with an Ocean Optics S2000-UV-Vis system. The average particle size, polydispersion index (PDI), and *Z* potential were measured with a dynamic light scattering (DLS) Zetasizer Nano ZS. Transmission electron microscopy (TEM) measurements were performed on a JEOL JEM-1230, working at 100 kV, obtaining images corresponding to the shape and size. XRD analysis of copper nanoparticles was conducted using an X-ray diffractometer Empyrean Alpha 1 of Malvern Panalytical., with Cu-K*α* X-rays of wavelength (*λ*) = 1.54056 Å, and infrared spectroscopy (FTIR) was performed with an IR Affinity-1 for the Copper-Soybean Chelate sample.

### 2.3. Antimicrobial Test of Nanoparticles

The following reactive materials were used: phosphate buffer Na_2_HPO_4_ and KH_2_PO_4_ (Fermont), Mueller-Hinton broth (BD Difco), and sodium chloride (NaCl, CTR Scientific). We analyzed the minimum inhibitory concentration (MIC) and the minimum bactericidal concentration (MBC) by the standard microdilution method (CLSI M100-S25 January 2015) [29], to determine the antimicrobial activity of the nanoparticles synthesized. The strains exposed to nanoparticles as the test control were *Escherichia coli* (ATCC 25922), *Staphylococcus aureus* (ATCC 29213), *Enterococcus faecalis* (ATCC 29212); later, three strains of marine origin were used, *Bacillus subtilis*, *Bacillus pumilus,* and *Bacillus altitudinis*. The bacterial concentration was standardized using the McFarland scale, and it was realized to an optical density of 0.2 at 568 nm (approximately 1 × 10^8^ CFU ml^−1^). The nanoparticles concentration employed against the strains was 2.5 mg·ml^−1^ for copper nanoparticles and 1.54*E* − 01 mg ml^−1^ for silver nanoparticles. Once the nanoparticles were dispersed, they were diluted with 50 *μ*l of Mueller–Hinton broth and 50 *μ*l of phosphate buffer previously inoculated with the tested strains at a concentration of 1 × 10^5^ CFU·ml^−1^. As the last step, the plate was incubated for 24 h at 36 ± 1°C.

### 2.4. Paint Preparation

For the preparation of antifouling paint, a commercial vinyl-acrylic paint Comex® Pro 1000 plus was used, which was prepared according to the method reported by Dominguez-Wong et al. [30]. 15% v/v of the Ag and Cu nanoparticles and copper-soybean chelate in solution was added to the paint. The concentration of the solutions added to the paint was 2.5 mg·ml^−1^ for copper nanoparticles and copper-soybean chelate and 1.54*E* − 01 mg·ml^−1^ for silver nanoparticles. This concentration is the same as that used for the antimicrobial assay of nanoparticles in solution. The paint without additives (control paint) was prepared according to the manufacturer's instructions, adding 15% water. To ensure good dispersion of the nanoparticles in the paint, each sample was homogenized using ultrasonic liquid processors (Sonics Vibra cell model CV33). The samples were processed for 10 minutes. An enough quantity of paint with additives was prepared for further evaluation over one year (initial time, 6 months, and 12 months). The glass samples used had a measure of 5 × 5 × 0.6 cm^3^, which were covered with three layers of paint for later evaluation. All the samples were evaluated in triplicate.

### 2.5. Evaluation of Color Variation in Paint Samples using the CIELAB76 Model

Colorimetric tests were carried out on the paints to define the color deviation using the CIELAB 1976 standard, which is one of the most widely used systems proposed by the Commission Internationale de l'Eclairage [31]. This system is defined by three coordinates, L^∗^ indicates the lightness and a^∗^ and b^∗^ are the chromatic coordinates [32].

  L^∗^ = lightness
  a^∗^ = red/green coordinates (+a indicates red, -a indicates green)
  b^∗^ = yellow/blue coordinates (+b indicates yellow, -b indicates blue)

The color difference or color variation is defined as the numerical comparison of a sample with a standard and is represented as delta (Δ*E*∗), and this color difference is determined according to Equation 1.

(1) $$\begin{array}{c} \Delta E^{*}=\;\sqrt{\Delta L^{*2}+\;\Delta a^{*2}+\;\Delta b^{*2}}. \end{array}$$

Color is an important property in the coatings and paints industry. The CIELAB76 calculation method is still the most commonly used. The tolerance or color acceptance is the maximum color difference admitted in products in relation to a standard, and in the coatings and paints industry, it is established that this variation must be in the range of 0.0 to 5.0 Δ*E*^∗^units. This deviation is industrially interpreted as follows [33–35]:

  Δ*E*^∗^< 1 Imperceptible
  Δ*E*^∗^ < 2 Minimum
  Δ*E*^∗^ < 3 Acceptable
  Δ*E*^∗^ < 5 Almost unacceptable
  Δ*E*^∗^ = 5 Unacceptable

The color coordinates of the paints (CIEL^∗^a^∗^b^∗^) were determined with the OceanOptics SpectraSuite® spectrometer software, and subsequently the color variation was calculated.

### 2.6. Determination of Cu and Ag Species Concentration in Paint

This test was performed to determine the real concentration of silver and copper species, once added to the paint, and correlate it with its initial concentration. The concentration of the Cu and Ag species contained in each paint sample was determined in an inductively coupled plasma atomic emission spectroscopy (ICP-OES iCAP 7000 series, Mod. ICAP 7400 Duo, Thermo Scientific). For this analysis, 25 ml of the acid mixture for digestion was added to 1 g of paint, it was heated for 2 hours at 200°C, and the recovered supernatant was made up to 25 ml with deionized water.

### 2.7. Antimicrobial Test of Paint

#### 2.7.1. Qualitative Test

The qualitative evaluation of the paint samples was carried out by adapting the Standard Practice for Determining Resistance of Plastics to Bacteria [36]. Different samples of Mueller–Hinton agar were inoculated at 45°C with three strains of marine origin, *B. subtilis, B. pumilus,* and *B. altitudinis*, with a final concentration of 5 × 10^5^ cells·ml^−1^. Once the agar was inoculated, fifteen Petri dishes were filled for each bacterial strain, each sample was tested in triplicate (control, control paint, paint + AgNP's, paint + CuNP's, and paint + copper-soybean chelate). Before the total solidification of the Mueller–Hinton agar, the painted glasses were placed on the surface of the agar. Finally, they were incubated at a temperature of 36°C ± 1°C for 24 h. The result of this test is through visual appreciation after incubation.

#### 2.7.2. Quantitative Test

For quantitative evaluation, the paint was evaluated based on the Japanese Industrial Standard JIS Z2801-ISO 22196, Antibacterial products—Test for antibacterial activity and efficacy [26]. According to the standard, the test was performed in triplicate for each paint sample, and the *R* value was calculated according to equation (2). Established as the antibacterial activity of an evaluated product, it can also be expressed as the decrease in bacterial growth in orders of magnitude. The JIS Z2801-ISO 22196 standard indicates that for a product to be classified as antimicrobial, the *R* value must be ≥2. The glass samples painted with the previously prepared paint were employed and untreated glasses were used as a positive control. The inoculum was prepared from pure cultures of the three strains of marine origin, *B. subtilis, B. pumilus,* and *B. altitudinis*, obtaining a concentration of 5 × 10^5^ cells·ml^−1^. The samples were inoculated with 200 *μ*l of the inoculum, and a 4 × 4 cm^2^ polyethylene film was placed on top of each one. Subsequently, serial dilutions of tenfold were made, and the method of pouring into a plate with Mueller–Hinton agar was carried out, for the recovery and counting of the colony forming units (CFU). Petri dishes were incubated at a temperature of 36°C ± 1°C.

(2) $$\begin{array}{c} R=\;\left[\log\left(\frac{B}{A}\right)-\log\left(\frac{C}{A}\right)\right]=\;\left[\log\left(\frac{B}{C}\right)\right], \end{array}$$

where *R* is the value of antimicrobial activity. *A* is the average of the number of viable cells recovered immediately (time *t* = 0 h) after inoculation of the sample without additive. *B* is the average of the number of viable cells recovered after 24 hours (time *t* = 24 h) after inoculation of the sample without additive. *C* is the average of the number of viable cells recovered after 24 hours (time *t* = 24 h) after inoculation of the sample with additive.

**Figure 2:**
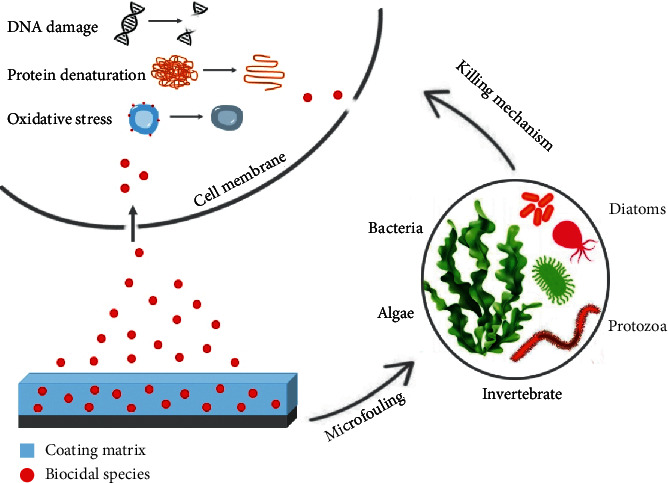
Schematic representation of the antifouling mechanism of paints.

All solutions used in this test were used at the appropriate salinity level and the principal components to simulate seawater conditions [37]. The purpose of this test is to simulate the first phase of colonization of a surface, which is characterized by the presence of algae, invertebrates, and bacteria followed by protozoa and diatoms. This film of organisms is called primary film or microfouling which is formed during the first hours of contact. The character of the resulting community is the one that gives way to macrofouling, up to development the biofilm [38, 39]. Therefore, if the first colonization can be inhibited, it is very likely that the formation of the biofilm will be prevented.  Figure 2 is a schematic representation of the possible antifouling mechanism by which paints can act against microorganisms.

## 3. Results

### 3.1. Characterization of Nanoparticles Solutions

#### 3.1.1. UV-Vis Analysis

**Figure 3:**
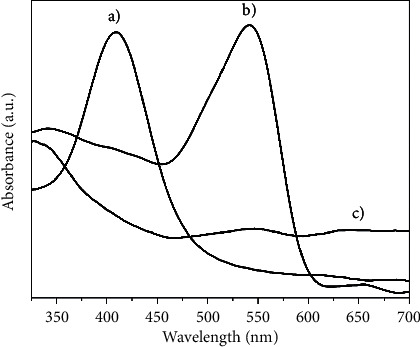
UV-Vis spectrum of (a) silver nanoparticles, (b) copper nanoparticles, and (c) copper-soybean chelate.

Figure 3 shows a group of UV-Vis spectra of the different electrolytic solutions. Figure 3(a) shows a typical absorption spectrum of the silver nanoparticles, and a narrow absorption band centered at 415 nm is shown [19, 40]. Figure 3(b) shows a defined absorption spectrum which proves the formation of the copper nanoparticles in the solution. The sample display a peak at around 543 nm [41, 42]. These peaks are due to the surface plasmon bands for the nanoparticles. Figure 3(c) corresponds to the sample of the copper-soybean chelate, where it is observed that the band associated with copper fades due to the formation of the soybean extract-copper complex [13]. The exact position of the resulting bands in the UV-Vis spectrum can change depending on the properties of the individual particles.

#### 3.1.2. DLS Analysis

**Figure 4:**
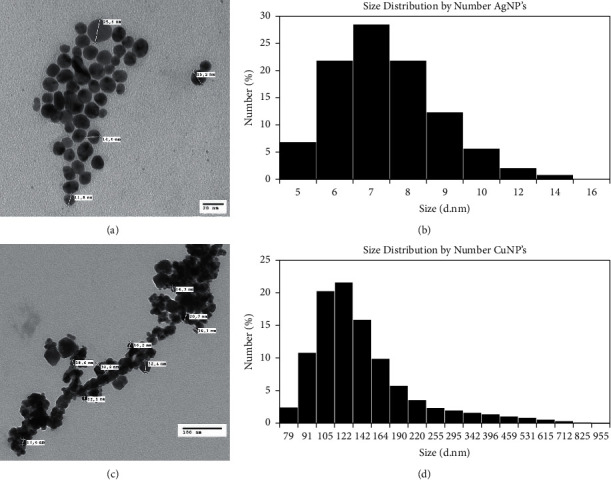
Dynamic light scattering measurements and TEM images. (a) TEM silver nanoparticles, (b) DLS silver nanoparticles, (c) TEM copper nanoparticles, and (d) DLS copper nanoparticles.

The nanoparticle solutions were analyzed by size using dynamic light scattering (DLS) as well as the polydispersion index (PDI) which is a measure of the size ranges present in the solution. The scale varies from 0 to 1, values close to zero indicate that the sample is monodisperse and values close to unity indicate that the sample has a great variety of sizes. *Z* potential was a measure too. This indicates if the surface charge of the nanoparticles is high enough to ensure the electrostatic stability of the suspension in the long term and avoid aggregation. The particle size distribution of colloidal solutions is shown in Figure 4.  Figure 4(b) corresponds to the measurement of silver nanoparticles where it shows an average size of 7 nm, and it is observed that the histogram has a narrow size distribution. The PDI obtained is 0.109 which determines the monodispersion of the sample, and the value obtained for the *Z* potential of the silver nanoparticles was −32.7 ± 11.5 mV indicates that the surface charge of the nanoparticles is high enough to ensure the electrostatic stability of their suspension, which prevents aggregation and contributes to the stabilization in long term. In Figure 4(d), it can be observed that the average particle size is 155 nm, obtained from the measurement of the copper nanoparticle sample. The PDI is 0.450, and the *Z* potential was −19.8 ± 12.4 mV, demonstrating the low stability causing the aggregation, which can be corroborated in the TEM images [42, 43].

#### 3.1.3. TEM Analysis

Figure 4 shows the TEM images of the nanoparticles prepared by electrolytic synthesis.  Figure 4(a) shows the silver nanoparticles, where it is shown the formation of the well-dispersed nanoparticles with spherical shape and uniform size, and the presence of particle aggregation is not observed [40].  Figure 4(c) corresponds to copper nanoparticles where the distribution is not uniform and have an irregular shape; in this case, the particles are larger and the presence of dense agglomerates or the tendency to cluster is observed.

#### 3.1.4. XRD Analysis

**Figure 5:**
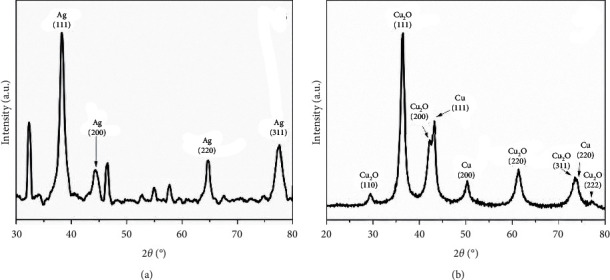
XRD pattern of nanoparticles. (a) silver nanoparticles and (b) copper nanoparticles.

Figure 5 corresponds to X-ray analysis of the nanoparticles. In Figure 5(a), there are four strong reflection peaks at 2*θ* values of 38.23°, 44.25°, 64.72°, and 77.45° corresponding to lattice planes of silver (111), (200), (220), and (311), respectively. All reflections correspond to FCC silver, and the lattice constant was determined at 4.07 Å. This result has highly matched with the standard powder diffraction card (JCPDS no. 04-0783) of Joint Committee on Powder Diffraction Standards. The XRD pattern of copper nanoparticles are shown in Figure 5(b)) with reflections at 2*θ* = 43.22°, 50.34°, and 73.95°, which corresponds to the crystalline plane (111), (200), and (220), respectively, of the face-centered-cubic structure (FCC), and the network parameter was 3.62 Å. It has been reported that for FCC materials, the reflection corresponding to plane (111) is the one with the highest intensity, which agrees with the measurements of the nanoparticles samples.

#### 3.1.5. Infrared Spectroscopy (FTIR)

**Figure 6:**
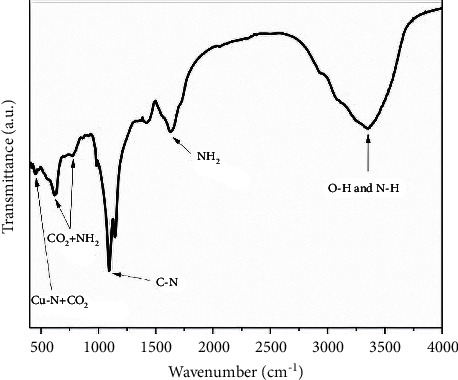
Infrared absorption spectra of copper-soybean chelate solution.

Figure 6 shows the infrared absorption spectrum of the copper-soybean chelate solution that presents bands in the range of 400 to 500 cm^−1^ which correspond to the formation of chelate, specifically the band at 460 cm^−1^ is associated to symmetrical stretching vibrations of Cu-N and CO_2_ bend wagging and the bands at 618 cm^−1^ and 775 cm^−1^ can be attributed to rocking vibrations of CO_2_ and NH_2_ due to the complex it forms with the amino acids [44]. At 1097 cm^−1^, the vibrations correspond to C-N, and the peaks at 1633 cm^−1^ are attributed to bend scissoring vibrations of NH_2_. After the 3000 cm^−1^ range (3290 to 3390 cm^−1^), the bands correspond to O-H and N-H vibrations.

### 3.2. Antimicrobial Assay of Nanoparticles

Table 1 shows the results for the antimicrobial test of the copper-soybean chelate, copper nanoparticles, and silver nanoparticles. The copper-soybean chelate compared to copper nanoparticles has a greater antibacterial activity when it was tested against *B. altitudinis* and *E. faecalis*, which is in accordance reported in the literature [13]. Concerning silver nanoparticles, the best antibacterial effect was observed against *S. aureus, B. pumilus,* and *B. altitudinis*, obtaining the same MIC value for the three strains, and in this particular assay, a higher efficacy was demonstrated for silver nanoparticles approximately ten times more effective compared to copper nanoparticles and copper-soybean chelate.

### 3.3. Color Variation of Paints using the CIELAB76 Model

The CIELAB system allows to define the color and the formula of color variation or color difference, and it manages to quantify the homogeneity of the color compared to a standard or reference according to the analyzed measurements. The values obtained from the paint samples to analyze the color variation are presented in Table 2, such as lightness (L^∗^), chromatic coordinates (a^∗^, b^∗^), and total color difference (Δ*E*^∗^). This test was reported in triplicate for each sample. The Δ*E*^∗^ levels calculated were less than 1 for the paint with copper nanoparticles and the paint with copper-soybean chelate and less than 2 for the paint with silver nanoparticles. These represent an imperceptible and minimum degree of color variation, respectively. The variation of the parameters in each coordinate with respect to the reference, and the Δ*E*^∗^ values indicate a good color homogeneity. The similarity between the samples is consistent with the use of the same paint additive.

### 3.4. Cu and Ag Species Concentration in Paint

The analysis of the paint using ICP-OES allowed us to determine the real concentration of the Cu and Ag species contained in each sample, and these results are shown in Table 3. For the control paint, a negative concentration calculation was obtained, which indicates that the paint used as the control does not contain Cu and Ag species in its initial composition. These values allow us to compare the percentage of Cu and Ag species present in the paint. If we compare them against the initial concentration of the added solutions (2.5 mg·ml^−1^ for copper nanoparticles and copper-soybean chelate and 1.54*E* − 01 mg·ml^−1^ for silver nanoparticles), the concentration determined by ICP-OES represents less than 1% of the initial concentration. In spite of low concentrations of Cu and Ag species, they can be correlated with a good antimicrobial activity, presented in the later sections.

### 3.5. Antimicrobial Assay of Paint

#### 3.5.1. Qualitative Assay

The samples evaluated by the Standard Practice for Determining Resistance of Plastics to Bacteria (control paint, paint + AgNP's, paint + CuNP's, and paint + copper-soybean chelate) presented the same degree of inhibition when in contact with the surface for all samples; however, they did not show an inhibition halo in the culture medium.

#### 3.5.2. Quantitative Assay—JIS Z2801-ISO 22196

The average of the count of recovered bacterial cells is presented in data of log_10_ CFU·ml^−1^ after 1 year of evaluation in Table 4, with test periods (initial time, 6 months, and 12 months). After evaluation, the control paint sample did not show any antibacterial activity, and the paint samples added with nanoparticles were compared in the percentage of activity and log_10_ CFU·ml^−1^ against the control paint. There is a difference of approximately two orders of magnitude (≈2 log_10_ CFU·ml^−1^) between all the paints with additive and the control paint for the three strains, which is equivalent to more than 99% inhibition in bacterial growth. Throughout the year of evaluation, there was an increase in the bacterial count (log_10_ CFU·ml^−1^) that indicates a slight decrease in the percentage of antibacterial activity of the paints.

## 4. Discussion

After the corresponding evaluations, we determined that the antifouling activity of the evaluated paint is attributed to the addition of the synthesized compounds.

TEM analysis results for copper nanoparticles (Figure 4(c)) showed the presence of agglomerates of particles. This is probably due to the solubility of copper nanoparticles in water, and this is one of the main disadvantages of these particles as well as their rapid oxidation [45–47]. The chosen method for efficient measurement of the nanoparticles was to disperse the powder by sonication just before depositing it on the TEM grid to avoid precipitation. XRD pattern (Figure 5(b)) showed additional peaks that are assigned to Cu_2_O, the network parameters are similar to those reported in the literature, and the formation of Cu_2_O indicates the partial oxidation of the nanoparticles. It can be indicated that the result obtained from the XRD pattern is as expected, contrasting to what has been reported by different authors for copper nanoparticles synthesized by different methods [41, 42, 46–48].

The difference in the results of the antimicrobial activity of the samples tested against the strains may be due to the structure of each bacterium: the cell wall is wider for Gram-positive strains, adhesion structures, virulence factors or pili type as well as the presence or absence of mobility structures or flagella [49]. Nevertheless in the case of silver nanoparticles, we observed that despite being two different genders of bacteria, *Bacillus* genus and *Staphylococcus* genus, the same MIC values were obtained in these strains, which can be explained by previous studies where they reported that through an atomic force microscope (AFM), the surface characteristics of Gram-negative and Gram-positive bacteria were studied and observed that species of the genus *Staphylococcus* showed a smooth surface practically equal to that of the surface area of species of the genus *Bacillus* [50]. Regarding paint, we can mention that the visible properties of the paint such as gloss and color were not modified when adding the nanoparticle solutions and the copper-soybean chelate. The qualitative analysis of the paint shows the absence of an inhibition halo in the medium, which could imply a strong adherence of the Cu and Ag species to the paint, that explains the absence of leaching of the biocidal species and only their degree of inhibition on contact with the surface.

**Figure 7:**
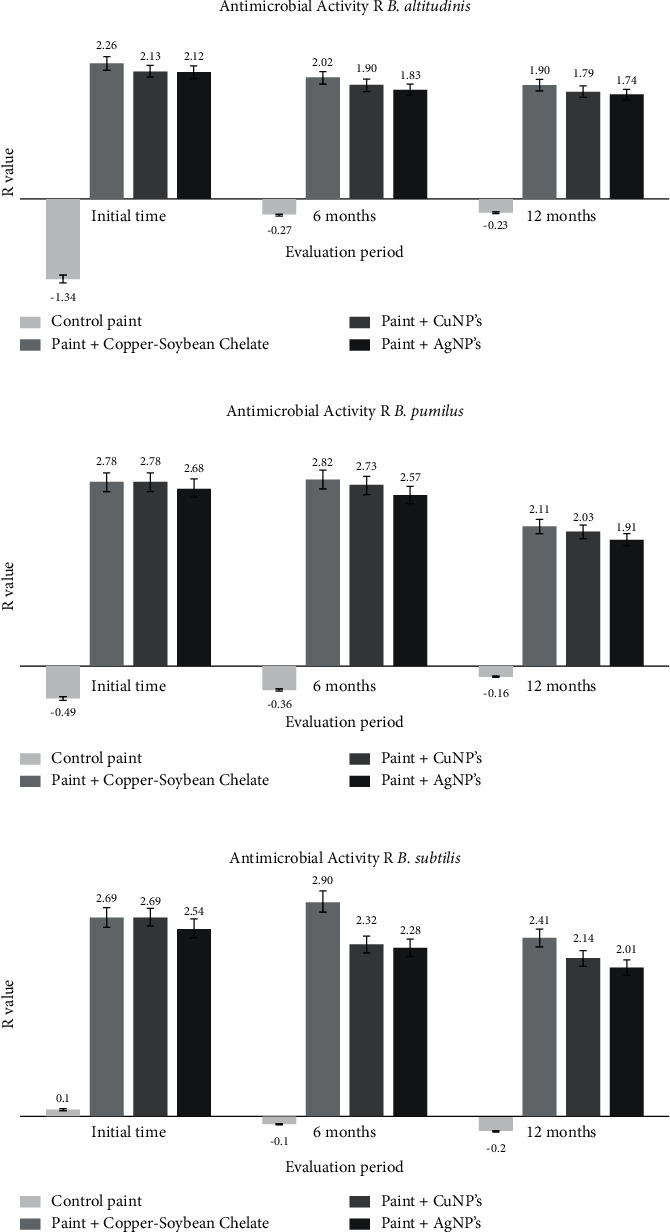
Antibacterial activity *R* of paint samples.

According to the Japanese Industrial Standard JIS Z2801-ISO 22196, a product is antimicrobial when the value of *R* ≥ 2.  Figure 7 shows the antibacterial activity (R) calculated in the three test times, where it is possible to appreciate the decrease of the R value and the behavior that each sample presented after one year of evaluation, and the graphics show that the paints with copper species (paint + copper-soybean chelate and paint + CuNP's) have a similar antibacterial activity in the initial time (R = 2.78, 2.69). After 12 months, the paint + copper-soybean chelate maintains an R value > 2 for B. pumilus and B. subtilis which is equivalent to 99.22% and 99.61% inhibition of bacterial growth, respectively; for B. altitudinis, the R value decreased (R = 1.90) with this paint. In the case of *B. altitudinis*, it is observed that after 6 months of evaluation for paint + CuNP's and paint + AgNP's, antimicrobial activity decreases and after 12 months, the three paint samples with additive have a value of *R* < 2 (*R* = 1.90, 1.79, and 1.74). For *B. pumilus*, the only sample that decreased its antimicrobial activity was paint + AgNP's at 12 months of evaluation (*R* = 1.91), and this sample is the one that showed the lowest antimicrobial activity for the three strains. The three paint samples with additive had a better antimicrobial activity when they were tested with *B. subtilis*, and there was a slight decrease throughout the year of evaluation; however, the *R* value remained at >2 (*R* = 2.41, 2.14, and 2.01) which is equivalent to more than 99% bacterial inhibition.

Correlating the results of the surface evaluation (JIS Z2801-ISO 22196) with the effective concentration of silver and copper species in the paint, obtained by ICP-OES, we can attribute, as mentioned above, the antimicrobial activity of the paints to the activity of the added biocidal species. What is important to highlight at this point is the high inhibitory capacity of the paints despite containing a very low concentration of Cu and Ag species compared to paints that have been used for antifouling purposes [51–53]. This can be translated into a great and novel option, getting effective antifouling additives at low concentrations, as well as the ease of access to these by means of a reproducible synthesis method.

The JIS Z2801-ISO22196 test show an important inhibition of bacterial growth, highlighting the results obtained for the paint added with copper-soybean chelate. We suggest that the presence of the organic part (amino acids) provided by the soybean extract in the chelate composition is the one that interferes in the effects of a high bacterial inhibition [13]. Biocidal species in ionic form have a specific interaction with amino acids due to the content of free sugar in the bacteria and the amino sugars present in the peptidoglycan chains [54] for that the copper-soybean chelate has the ability to attract microorganisms and cause their cell death because the ions it contains has a major affinity than the nanoparticles and it is easier for them to penetrate the cell membrane of the bacteria. For this reason, despite having a different concentration than the copper and silver nanoparticles in the paint, it can resemble and even improve its ability to inhibit bacteria. It is important to mention that this work is the first phase of the study, and the results obtained will allow us to continue with the investigation of the sample that produced the best results (copper-soybean chelate), to carry out the growth of a bacterial biofilm and the corresponding study of the interactions between this and the paint sample, in order to establish whether the second phase of colonization of a surface can be inhibited as well as to determine the concentration of ions released from the additive after interacting with the biofilm.

Nowadays, the use of compounds and nanostructures of different materials to determine their antimicrobial, antifungal, antifouling, and other properties is increasing; however, the biochemical process or the mechanism by which they act on microorganisms has not been completely elucidated, and different routes of action have been proposed for these biocidal species to cause cell or bacterial death.

Talking about the ionic form of silver or silver nanoparticles, the biochemical mechanisms involved can be several, such as the damage of the cell wall produced by silver as well as the accumulation of silver in the bacterial membrane. It is believed that Ag breaks the permeability of the outer membrane of the bacteria and affects their peptidoglycan chains, causing the leakage of cellular materials [55]. This leads to the inhibition of respiratory chain dehydrogenases, while some proteins and phospholipids induce membrane collapse and subsequently cell death [54].

It is well known that transition metals are toxic to bacteria, and although copper is an essential trace element for bacterial cells because it is involved in the synthesis of metalloproteins, such as electron-transport proteins, its ionic form is considered toxic at higher concentrations. A low molecular-weight compound can bind to available copper ions and thus alter their biological function [56]. The probable biochemical process involved in the antibacterial activity of copper species begins with the absorption of Cu ions by the bacteria, which adhere to the bacterial cell wall that is negatively charged and cause the breakdown, followed by a cascade of events for the reduction of these ions. Subsequently, these particles are released from the cell using the bacterial efflux system, that causes protein denaturation and cell death [57, 58]. Once copper enters the bacterial cell, it can bind to deoxyribonucleic acid molecules causing rapid DNA degradation, followed by a reduction in bacterial respiration as well as the inhibition of bacterial membrane cytochromes. Absorption of copper ions by bacterial cells also disrupts important biochemical processes [57, 59, 60].

## 5. Conclusions

This work conferred a significant improvement to the paint samples employing nanostructured compounds as additives and studying their antifouling capacity, which showed a high percentage of bacterial inhibition against *Bacillus* species, in which copper-soybean chelate presented the best antifouling activity. Our results suggest that using copper species and AgNP's can increase the resistance to biofouling of surfaces.

## Figures and Tables

**Table 1:** Minimum inhibitory concentration (MIC).

	MIC nanoparticles (mg·ml^−1^)		
Bacterial strains	Sample		
	Copper-soybean chelate	Copper nanoparticles	Silver nanoparticles
	(2.5 mg·ml^−1^)		(1.54*E* − 01 mg·ml^−1^)
*E. coli* ATCC 25922	0.62	1.25	9.7*E* − 03
*S. aureus* ATCC 29213	0.62	2.5	4.8*E* − 03
*E. faecalis* ATCC 29212	0.31	1.25	3.9*E* − 02
*B. subtilis*	2.5	1.25	9.7*E* − 03
*B. pumilus*	2.5	1.25	4.8*E* − 03
*B. altitudinis*	0.62	1.25	4.8*E* − 03

**Table 2:** Color variation of the paint samples.

Color variation of paints using the CIELAB76 model					
Sample	CIE L^∗^	CIE a^∗^	CIE b^∗^	Δ*E*^∗^	Color acceptance
Control paint reference	99.81	0.3	0.2		
Paint + copper-soybean chelate					
1	99.41	0.2	0.8	0.73	Imperceptible
2	99.41	0.1	0.8	0.75	
3	99.60	0.2	0.9	0.74	
Paint + CuNP's					
1	98.95	−0.1	0.5	0.99	Imperceptible
2	99.15	−0.1	0.5	0.83	
3	99.01	0.1	0.4	0.85	
Paint + AgNP's					
1	99.6	−0.1	1.6	1.47	Minimum
2	99.8	−0.2	1.5	1.39	
3	99.8	−0.2	1.4	1.3	

**Table 3:** Cu and Ag species concentration in paints.

	mg Cu g^−1^ paint	mg Ag g^−1^ paint
Control paint	−3.46*E* − 05	−6.34*E* − 04
Paint + copper-soybean chelate	1.14*E* − 02	—^a^
Paint + CuNP's	9.07*E* − 03	—^a^
Paint + AgNP's	—^a^	6.94*E* − 04

^a^No evidence of Cu or Ag species was found in these samples.

**Table 4:** Bacterial cell count recovered from JIS Z2801-ISO 22196 (log_10_ CFU·ml^−1^).

Bacterial cell count recovered (log_10_ CFU·ml^−1^)										
Bacterial strains		*B. altitudinis*			*B. pumilus*			*B. subtilis*		
Sample		Period								
		Initial time	6 months	12 months	Initial time	6 months	12 months	Initial time	6 months	12 months
Control paint	1	5.34	5.43	5.80	6.81	6.83	6.79	7.67	7.91	7.83
	2	5.36	5.45	5.81	6.85	6.82	6.78	7.70	7.90	7.83
	3	5.32	5.43	5.79	6.80	6.84	6.78	7.69	7.88	7.83
	Average	**5.34**	**5.44**	**5.80**	**6.82**	**6.83**	**6.78**	**7.69**	**7.90**	**7.83**
	Std dev	±**0.02**	±**0.01**	±**0.01**	±**0.03**	±**0.01**	±**0.01**	±**0.03**	±**0.01**	**0**
	% bacterial inhibition	**0**	**0**	**0**	**0**	**0**	**0**	**0**	**0**	**0**

Paint + CS-Ch	1	3.18	3.40	3.91	4.11	4.00	4.69	5.00	5.00	5.43
	2	3.08	3.28	3.89	4.00	4.00	4.68	5.00	5.00	5.38
	3	2.95	3.53	3.90	4.00	4.04	4.65	5.00	5.00	5.46
	Average	**3.07**	**3.40**	**3.90**	**4.04**	**4.01**	**4.67**	**5.00**	**5.00**	**5.42**
	Std dev	±**0.11**	±**0.13**	±**0.01**	±**0.07**	±**0.02**	±**0.02**	**0**	**0**	±**0.04**
	% bacterial inhibition	**99.45**	**99.05**	**98.75**	**99.83**	**99.85**	**99.22**	**99.79**	**99.87**	**99.61**

Paint + CuNP's	1	3.23	3.54	4.00	4.11	4.11	4.76	5.00	5.32	5.72
	2	3.34	3.56	4.00	4.00	4.18	4.77	5.00	5.73	5.68
	3	3.00	3.49	4.02	4.00	4.00	4.74	5.00	5.57	5.70
	Average	**3.19**	**3.53**	**4.01**	**4.04**	**4.10**	**4.76**	**5.00**	**5.54**	**5.70**
	Std dev	±**0.17**	±**0.03**	±**0.01**	±**0.07**	±**0.09**	±**0.02**	**0**	±**0.21**	±**0.02**
	% bacterial inhibition	**99.25**	**98.75**	**98.39**	**99.83**	**99.81**	**99.06**	**99.79**	**99.52**	**99.27**

Paint + AgNP's	1	3.15	3.51	4.05	4.28	4.20	4.86	5.15	5.59	5.83
	2	3.28	3.67	4.06	4.00	4.30	4.83	5.00	5.63	5.82
	3	3.23	3.62	4.07	4.08	4.26	4.93	5.26	5.61	5.83
	Average	**3.22**	**3.60**	**4.06**	**4.12**	**4–25**	**4.88**	**5.13**	**5.61**	**5.83**
	Std dev	±**0.07**	±**0.09**	±**0.01**	±**0.14**	±**0.05**	±**0.05**	±**0.13**	±**0.02**	±**0.01**
	% bacterial inhibition	**99.24**	**98.52**	**98.18**	**99.79**	**99.73**	**98.76**	**99.71**	**99.48**	**99.02**

^*∗*^Paint + CS-Ch = paint + copper-soybean Chelate. Bold values are the outstanding results of the samples after being evaluated in triplicate.

## Data Availability

The data that support the results of this study are available on request from the corresponding author.
